# The Uniform Certificate—II

**Published:** 1908-04-11

**Authors:** 


### Nursing Administration.

### THE UNIFORM CERTIFICATE.—II.

It has been abundantly proved that people do not show any disposition to embrace the opportunity of entering their names on an official list unless compelled thereto by penal legislation, or bribed by the offer of unmistakable advantages. The good ones find that they can get on very well without this formality. The bad ones, who just manage to scrape in, are in reality no better off. After an admirably organised attempt on the part of the teaching profession to maintain an official List of Teachers the design has fallen through, not because it was not an excellent scheme, but from the apathy of the profession. There is a close parallel between teaching and nursing, dissimilar as these branches of the public service may appear at first sight. Both arts are taught by continual exercise, on the "corpus vile" of pupil and patient. In both cases the training grounds are independent public bodies, of a self-governing character, existing not solely or mainly in order to train teachers and nurses; in both cases unfettered save by public opinion and the honour of the establishment.

The big public schools of the country, endowed by the liberality of former generations, correspond closely in constitution and freedom to the big clinical hospitals. The grammar schools partially endowed and embued with a healthy spirit of rivalry, one with another, answer to the provincial and other general hospitals depending largely on local support; the newer schools under the control of companies or private individuals answer to institutions of a special character altogether dependent on popular favour. All these establishments have been levelled up in the course of the last few decades, not by legislation, but by the simple process of setting a standard which has gradually come to be accepted by every school worthy of the name. The standard set by the Oxford and Cambridge examiners, subsequently amalgamated into a joint board, has been conclusively, if gradually, adopted throughout the country. The growth of examination has proved not merely a stimulus to pupils preparing to be teachers, it has been the best possible aid to the conduct of the whole establishment. And we find both public and private schools invoking the aid of outside examiners to report on the general tone and administration of the classes, and to proffer recommendations.

What has thus been gradually effected in the direction of increased efficiency in education without State intervention is plainly within the reach of the voluntary hospitals of this country. The agreement of ten or twelve influential hospitals, for the purpose of issuing a uniform certificate, would draw irresistibly others in its train. Such a certificate, grading nurses by a fixed scale of marks, implies the existence of an examination common to all, and the scale of marks could hardly be adjusted with any justice, unless awarded by outside examiners. We believe expert teachers, of nurses charged with the duty of visiting hospitals for the purpose of examining probationers at the conclusion of their training, would be welcomed in countless institutions where a strong desire exists to provide good training, and to gain a more complete understanding of the vital points in the probationer's course. Such knowledge is expert work. The matron ought not, as often happens, to have the entire responsibility thrown upon her. She may quite conceivably have been trained herself in a good training school, and yet may find herself hampered in every possible way in new surroundings in her endeavour to give her probationers the amount of teaching she knows they ought to have. Had she the support of an outside examiner in instituting reforms she would hold a far stronger position. Consultation with the examiner—we are assuming that so far as practical work is concerned the examiner would be herself a trained nurse—would be of the greatest possible aid in showing matrons of small institutions the best methods of adapting the means at their disposal to the desired end, and the general level of nursing education would benefit no less than the internal efficiency of the establishment.

The principle of compulsion has been rejected with absolute distinctness by every hospital in the kingdom. But the same desire for the honour and increased prosperity of the establishment which induces the managers of schools to invite outside experts to examine and report on their progress would unquestionably work with the governors and officials of voluntary institutions for the care of the sick, and the additional reputation conferred on those who were united in the cause of nursing education by the fact of their aiming at a common standard would act as a stimulus on those who were indifferent or negligent. It is comparatively easy both in teaching and in nursing to set up a barrier and declare that none shall pass save those who have strength to take the leap. A few will take it, the majority will walk round another way, declaring, "I could if I would, but I won't." The really important thing is so to train the physique that an increasing number have the requisite strength to pass the test. For that we must look to the training schools. Nurses are unhappily so regardless of their future, so bent upon immediate advantage, even if it be the mere saving of a guinea by neglecting to put their names down on a list, that it is to those who train them that we must look for security in the interests of the public that all who enter the profession are properly equipped for their work. We may wish the new measure which Lord Balfour of Burleigh is introducing all success in its limited and very difficult task of gathering together the names of those who are engaged in nursing the sick. But we must not look to it for help in raising the standard of nursing education, for that is a matter which lies altogether outside its scope. Is it not time that some agreement was reached on this all-important question?

